# Inflammation in heart failure: pathophysiology and therapeutic strategies

**DOI:** 10.1007/s00011-023-01845-6

**Published:** 2024-03-28

**Authors:** Jacinthe Boulet, Vikas S. Sridhar, Nadia Bouabdallaoui, Jean-Claude Tardif, Michel White

**Affiliations:** 1 Department of Medicine, Division of Cardiology, Montreal Heart Institute, Université de Montréal, Montreal, QC Canada; 2 Department of Medicine, Division of Nephrology, University Health Network, Toronto, ON Canada; 3 Department of Medicine, Division of Cardiology, Montreal Heart Institute, Université de Montréal, 5000 Belanger Street, QC H1C 1C8 Montreal, Canada

**Keywords:** Heart failure, Inflammation, Cardiomyopathy, Immune system, Cytokines

## Abstract

A role for inflammation in the development and progression of heart failure (HF) has been proposed for decades. Multiple studies have demonstrated the potential involvement of several groups of cytokines and chemokines in acute and chronic HF, though targeting these pathways in early therapeutic trials have produced mixed results. These studies served to highlight the complexity and nuances of how pro-inflammatory pathways contribute to the pathogenesis of HF. More recent investigations have highlighted how inflammation may play distinct roles based on HF syndrome phenotypes, findings that may guide the development of novel therapies. In this review, we propose a contemporary update on the role of inflammation mediated by the innate and adaptive immune systems with HF, highlighting differences that exist across the ejection fraction spectrum. This will specifically be looked at through the lens of established and novel biomarkers of inflammation. Subsequently, we review how improvements in inflammatory pathways may mediate clinical benefits of existing guideline-directed medical therapies for HF, as well as future therapies in the pipeline targeting HF and inflammation.

## Introduction

A role for inflammation in the development and progression of heart failure (HF) has been proposed for more than three decades. While several analyses have since demonstrated the potential involvement of several groups of cytokines and chemokines in acute and chronic HF, targeting these pathways in therapeutic trials was initially not successful. These early studies served to highlight the complexity and nuances of how pro-inflammatory pathways contribute to the pathogenesis of HF. More recently, a few studies have demonstrated potential benefits of targeting inflammation on clinical outcomes in patients with heart disease. In this review, we first discuss the role of inflammation mediated by the innate and adaptive immune systems in HF, highlighting differences across the HF ejection fraction (EF) spectrum. This is specifically looked at through the lens of established and novel HF and inflammatory biomarkers. Subsequently, we review how improvements in inflammatory pathways may mediate clinical benefits of existing guideline-directed medical therapies (GDMT) for HF, as well as therapies and strategies targeting inflammation in HF.

### Inflammation across the HF spectrum

Inflammation contributes to the development of HF across the EF spectrum and, through an array of mechanisms, HF itself promotes a chronic inflammatory state, two interconnected, and bidirectional processes. The concept of “inflamm-aging,” describing the state of chronic low-grade inflammation occurring in older organisms, has been proposed as a driver of age-related chronic disease including CV disease and ischemic cardiomyopathy (CMP) [1]. Inflammation is highly prevalent across acute and chronic HF, from reduced to preserved EF, as demonstrated in several large cohorts [2–4]. Additionally, a broad range of biomarkers associated with vascular injury and inflammation in stable advanced HF patients awaiting heart transplant were found to be significantly increased, remaining elevated after transplantation [5].

Biomarker profiles differ across the spectrum of EF. In a network meta-analysis of biomarker profiles in HF, Tromp and colleagues reported that HFrEF patients predominantly exhibited a “cardiac stretch” profile with biomarkers related to cellular proliferation and metabolism [6]. However, most biomarkers associated with HFpEF were related to cardiac inflammation and patients showed a more heterogeneous range of biomarkers, much like their diverse clinical profiles. Patients with HF and mid-range ejection fraction (HFmrEF) showed an intermediary profile with abnormal biomarker levels of both cardiac stretch and inflammation. It may be that while there is an underlying group of mechanisms that may be common across the EF range, the contribution of inflammation may be more specific and predominant at one end of the spectrum [7]. The inflammatory/pro-fibrotic paradigm describes one such group of mechanisms believed to drive disease pathogenesis in HFpEF [8]. Comorbidities such as obesity, diabetes, hypertension, and/or chronic kidney disease promote systemic inflammation that leads to a sequence of events including abnormal laminin–titin interactions, cardiomyocyte expression of inducible nitric oxide, and failing unfolded protein response within the myocardium. This culminates in myocardial fibrosis, hypertrophy, high diastolic left ventricular stiffness, and clinical HF [9]. Identification of mechanisms unique to HF phenotypes, like the inflammatory/pro-fibrotic paradigm in HFpEF, may help better organize the HF syndrome phenotypes by the predominant underlying pathophysiology, slowly moving away from the current classification solely based on ejection fraction, while guiding the development of novel therapies targeting phenotype-specific mechanisms.

### Selected biomarkers in the context of inflammation and heart failure

**Fig. 1:**
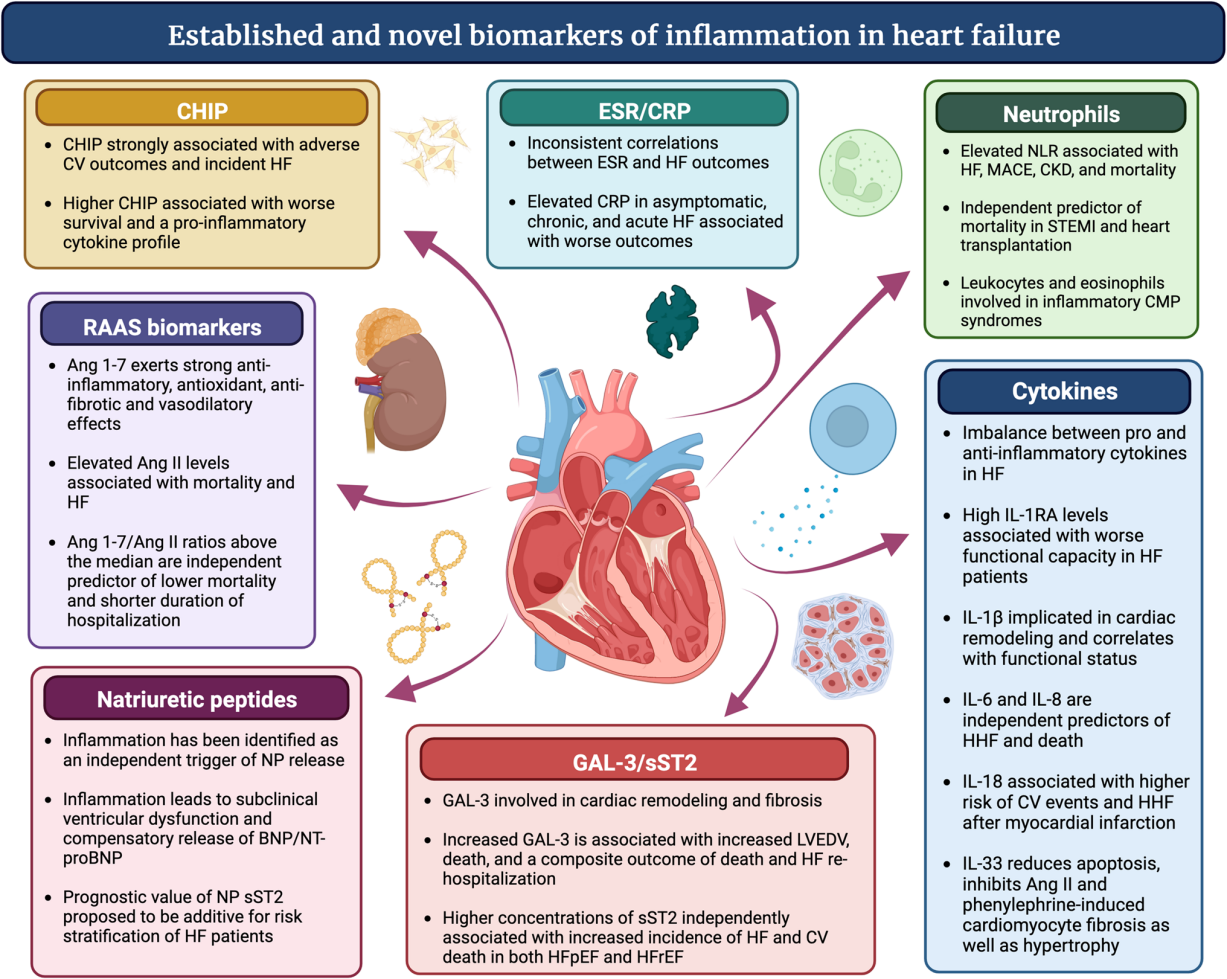
Established and Novel Biomarkers of Inflammation in Heart Failure. *Ang* angiotensin, *BNP* brain natriuretic peptide, *CKD* chronic kidney disease, *CRP* C-reactive protein, *ESR* erythrocyte sedimentation rate, *GAL-3* galectin-3, *HF* heart failure, *HFpEF* heart failure with preserved ejection fraction, *HFrEF* heart failure with reduced ejection fraction, *HHF* heart failure hospitalization, *IL* interleukin, *IL-1 RA* interleukin-1 receptor antagonist, *LVEDV* left ventricular end-diastolic volume, *MACE* major adverse cardiac events, *NLR* neutrophil-to-leukocyte ratio, *NP* natriuretic peptide, *sST2* soluble suppression of tumorigenesis-2, *STEMI* ST-elevation myocardial infarction, *CMP* cardiomyopathy

The contribution of systemic and local inflammation to the pathogenesis of HF has been previously reviewed and is beyond the scope of this manuscript [10, 11]. In this section, we will review biomarkers of inflammation (Fig. 1), assessing whether changes in these biomarkers reflect pathways that mediate changes in clinical status and modify prognosis. Additionally, we will comment on the link between established and novel biomarkers of HF and inflammatory pathways.

#### C-reactive protein

CRP is an acute phase pro-inflammatory cytokine predominantly synthesized by hepatocytes in response to interleukin (IL)-6 signaling. It has emerged as a potential risk predictor of adverse outcomes in HF patients. In patients without HF or ischemic heart disease at baseline, Vasan et al. assessed the relationship between CRP and other markers of inflammation and the incidence of HF events [12]. After adjusting for traditional risk factors, subjects with baseline CRP levels ≥ 5 mg/dl had a 2.8-fold increased risk of HF events (*p* = 0.02). The elevation of other inflammatory markers in addition to CRP further increased this risk. Pellicori and colleagues performed a similar analysis in patients with chronic HF referred to a HF clinic [13]. Higher baseline levels of hs-CRP independently predicted greater all-cause and CV mortality, after adjusting for age, symptom severity, creatinine, NT-proBNP and, in the case of CV mortality, baseline ejection fraction. It is unclear if targeting inflammatory pathways that determine CRP levels improve clinical outcomes in HF patients, particularly when considering side effects of these therapies. Additionally, it remains to be determined if some of the benefits of established HF therapies are mediated by reductions in CRP levels.

#### Uric acid

Uric acid has been recognized as a marker of systemic inflammation and as an adverse prognostic marker in HF [14, 15]. Hyperuricemia is also common, with a National Health and Nutrition Examination Survey reporting a prevalence in HF patients of up to 50% [16]. Hyperuricemia has been associated with elevated circulating levels of interleukins and TNF-alpha [14]. Through several proposed mechanisms, uric acid may also contribute to oxidative stress at the organ level. Hyperuricemia can up-regulate xanthine oxidase activity leading to increased generation of reactive oxygen species (ROS), which in turn contributes to cardiac hypertrophy and fibrosis [17].

#### Angiotensin-converting enzyme 2 and renin–angiotensin–aldosterone system biomarkers

Angiotensin II (Ang II) has pro-inflammatory and pro-fibrotic properties and has been clearly implicated in the pathogenesis of HF, and Ang II levels may remain elevated despite optimal GDMT. Angiotensin-converting enzyme 2 (ACE2) breaks down Ang II to angiotensin 1–7 (Ang 1–7) which exerts strong anti-inflammatory, antioxidant, anti-fibrotic, and vasodilatory effects. Elevated Ang II levels in the face of optimal RAS blockade with ACE inhibitors is associated with mortality and HF [18]. Increased human plasma ACE2 activity is also associated with increased HF severity and lower LVEF [19]. Wang and colleagues measured plasma angiotensin peptides in a diverse cohort of HF patients including clinic and emergency room patients with HFpEF and HFrEF that were followed for a median of 5.1 years [20]. In an adjusted multivariate analysis, Ang 1–7/Ang II ratios above the median were an independent predictor of lower mortality and shorter duration of hospitalization. Individual levels of Ang 1–7 or Ang II did not have a prognostic significance, in what the study authors describe as a reflection of the dynamic state of the RAS and its detrimental effects increased Ang II without a balancing increase in Ang 1–7 levels. Aldosterone additionally contributes to mechanistic pathways that promote HFrEF by modulating the effect of Ang II on plasminogen activator inhibitor-1, which in turn promotes oxidative stress and organ-level fibrosis.

#### Neutrophils

Neutrophils play an important role in the progression of CV diseases, including the mediation of tissue damage and cardiac remodeling [21]. In patients with advanced HF, the neutrophil-derived inflammation response at rest, after stimulation, and after attenuation by immunosuppressive agents have been investigated [22]. The isolation of neutrophils in these patients demonstrated a profound decrease in VEGF and Il-8 release after the use of pro-inflammatory agents, whereas IL-1RA was found to be elevated. Other studies have investigated the relevance of the neutrophil-to-leukocyte ratio (NLR) which has been shown to be strongly associated not only with HF and increased mortality but also with major CV events, HF hospitalizations, and chronic kidney disease in elderly patients [23, 24]. In a secondary analysis of the BIOSTAT-CHF study composed of patients with worsening or new-onset HF (HFrEF and HFpEF), baseline NLR was independently associated with the primary outcome of time to all-cause mortality or HF hospitalization, irrespective of EF [25]. Consistent with the assumption that the NLR is a wide-available marker of inflammation, NLR was also strongly associated with other well-established inflammatory biomarkers including IL-6 and sST2, as well as NT-proBNP. Importantly, not only neutrophils but also leucocytes and eosinophils contribute through distinct inflammatory pathways to the pathogenesis of HF, such as seen with inflammatory cardiomyopathy syndromes [26]. The role of lymphocyte is discussed in more details in the diuretic and decongestant therapies section below. Despite the evidence that neutrophils play an important role in the pathogenesis of HF and their established association with HF severity and overall mortality in patients with different HF etiologies [27], therapies targeting neutrophils remain limited.

#### Natriuretic peptides

Natriuretic peptides (NP) including B-type natriuretic peptide (BNP) and N-terminal pro BNP (NT-proBNP) are typically considered neurohumoral biomarkers, although inflammation has been shown to be an independent trigger of NP release [28]. In one cohort of participants, IL-6 levels independently and positively correlated with NT-proBNP levels after 4 years of follow-up. In another cohort of healthy individuals, administration of lipopolysaccharide, a pro-inflammatory stimulus, significantly increased median NT-proBNP levels. McKechnie et al. further explored this inter-relationship between inflammation and NP in a secondary analysis of the British Regional Heart Study where inflammation was associated with HF when adjusted for traditional risk factors [29]. However, additional adjustment for NT-proBNP eliminated much of the relationship between inflammation, measured by IL-6 and CRP levels, and HF events in an older male population. The authors suggest that NP may therefore explain much of the association between inflammation and HF events, with inflammation leading to subclinical ventricular dysfunction and compensatory release of BNP/NT-proBNP. However, it remains to be established whether there is role for NP in tracking inflammation in clinical practice where it may be more difficult to tease apart the various mechanisms that influence levels of this biomarker.

#### Inflammatory cytokines

The HF state is characterized by an imbalance between pro- and anti-inflammatory cytokines [30]. The predominance of pro-inflammatory cytokines has been correlated with increased severity of HF [31]. At the cellular level, these cytokines are mostly released by neutrophils and contribute to cardiomyocyte apoptosis and matrix metalloproteinase activation, leading to cardiac remodeling and alterations in heart function [32, 33]. Many pro- and anti-inflammatory cytokines have been shown to play a significant role in HF, notably IL-1, IL-6, IL-8, IL-18, IL-1RA, and IL-33.

IL-1β is an important pro-inflammatory cytokine regulating downstream factors in the immune response and promoting the migration of leukocytes to site of tissue injury [34]. In the setting of HF, IL-1β has been implicated in adverse cardiac remodeling. In animal models, inhibition of IL-1β has been shown to suppress progression to overt HF [35]. In humans, regardless of etiology, IL-1β has been shown to be proportional to NYHA functional status [36]. Similar to IL-1, IL-18 activation necessitates the activation of the NLRP3 inflammasome and has been associated with higher risk of cardiovascular events and hospitalization for congestive HF in post-myocardial infarct patients [37]. Another important pro-inflammatory cytokine found to be elevated in HF patients is IL-6. IL-6 has been independently associated with HF hospitalization and death [38]. Within the general population, elevated IL-6 levels were also demonstrated to independently predict the development of HFpEF [39]. Like IL-6, IL-8 levels are also elevated in patients with HF compared to healthy controls and associated with a worse clinical prognosis [27, 33, 40]. A member of the anti-inflammatory interleukin family, IL-1RA has also been found to be elevated in patients with HFrEF with or without diabetes compared to healthy controls [27]. Among anti-inflammatory cytokines, IL-33 has been found to be depressed in HF patients compared to healthy controls [34], and further down-regulated along with ST2-L in patients with decompensated HF awaiting LVAD support. Treatment with LVAD subsequently increased IL-33 and ST2-L cardiac expression, consistent with the proposed role this pathway plays in mechanical cardiac stress [41].

#### Soluble toll-like receptor 2

Another biomarker with potential for future clinical use in the management of HF is the soluble suppression of tumorigenesis (sST2), a member of the IL-1 receptor family [42]. Initially classified as a biomarker reflecting myocardial stress [43], sST2 is now recognized as a marker of inflammation and fibrosis, released in response to vascular congestion as well as inflammatory and pro-inflammatory stimuli [44]. At the cellular level, ST2-L, a trans-membrane receptor, binds to IL-33 which is activated after exposure to pathogens, injury-induced stress, and necrosis [45]. IL-33 binds to ST2-L and is proposed to possess cardioprotective properties including reducing apoptosis [46], as well as inhibiting Ang II and phenylephrine-induced cardiomyocyte fibrosis and hypertrophy [47]. The main clinical application of sST2 in HF is outcome prediction, and its prognostication value is additive to NP [44, 45]. Increased incidence of HF and CV death is associated with higher concentrations of sST2, both in HFpEF and HFrEF [48, 49], and this association was found to be independent of standard CV risk factors as well as other markers of inflammation including CRP and NP [50]. In chronic HF, sST2 levels hold a stronger prognostic value than Gal-3 [51] which has in turn been reported to be more predictive of acute HF [52]. Aimo et al. reviewed the clinical and prognostic significance of sST2 in HF and outlined that, similar to NPs, sST2 measurements are accurate and affordable allowing enhanced risk stratification in addition to usual clinical and biochemical evaluation of HF patients [44]. However, the optimal timing, measurement, and clinical use of sST2 level remain to be clarified.

#### Galectin-3

Galectin-3 (GAL-3) is a member of the galectin family of lectins that bind cell surface and intra-cellular ligands and are involved in various intra-cellular signaling pathways [53]. GAL-3 was first identified as a potential biomarker of decompensated HF in an animal model by Sharma and colleagues [54]. The authors put forth that GAL-3 was directly involved in adverse cardiac remodeling and therefore a potential therapeutic target. Besler et al. studied the relationship between GAL-3, inflammation, and fibrosis, revealing contrasting correlations depending on the etiology of CMP [55]. In endomyocardial biopsies obtained from patients with non-ischemic dilated CMP and inflammatory CMP, myocardial GAL-3 expression positively correlated with inflammatory cell count but negatively correlated with fibrosis in inflammatory CMP. Conversely, a positive correlation between myocardial GAL-3 and fibrosis was observed in dilated CMP.

The potential for GAL-3 as a clinical biomarker has also been shown in a number of human studies. In a study of 232 patients with NYHA class III or IV chronic HF, circulating GAL-3 was an independent predictor of mortality during a 6.5-year follow-up period after adjusting for the severity of HF (determined by NT-proBNP levels) and kidney function [56]. In a subsequent study of this cohort, GAL-3 was also positively correlated with an increase in left ventricular end-diastolic volume in a multivariate analysis [57]. Another study compared multiple plasma biomarkers including GAL-3 and NT-proBNP in 209 patients diagnosed with acute HF [58]. While the natriuretic peptide remained the superior biomarker for diagnosing HF, GAL-3 was the strongest predictor of death and the composite of death and rehospitalization with HF. The rationale and evidence for the use of GAL-3 as a marker of inflammation/fibrosis and as a prognostic marker in acute and chronic HF are certainly compelling though larger studies are required.

#### Growth/differentiation factor 15

Growth/differentiation factor 15 (GDF-15) is a member of the transforming growth factor-β superfamily who plays an important role in cell proliferation and differentiation, as well as in tissue homeostasis [59]. GDF-15 concentrations may increase in response to inflammatory or hypoxic stimuli, oxidative stress, and tissue injury. Studies have shown the predictive role of GDF-15 across an array of CV diseases, including an independent prognostic role in stable patients with chronic HFrEF [60–62]. Despite the finding that a 20% increase in GDF-15 levels was associated with a statistically significant higher risk of mortality and poor CV outcomes in the PARADIGM-HF study population, GDF-15 levels were not affected by sacubitril–valsartan therapy [63]. The ability to predict poor CV outcomes was found to be independent of other biomarkers of significance in HF, namely, NT-proBNP and high-sensitivity troponins. The role of GDF-15 in HF patients needs to be better defined and additional studies would likely help identifying its potential use for the diagnosis and management of HF patients [64].

#### Clonal hematopoiesis of indeterminate significance

Clonal hematopoiesis of indeterminate potential (CHIP) is a hematologic disorder characterized by age-related acquisition of mutations in hematopoietic stem cells [65], leading to a clonal myeloid cell population that is associated with increased systemic inflammation [66]. The presence of CHIP is strongly associated with adverse cardiovascular outcomes, including incident HF [67] and increased death/HF hospitalization in patients with chronic HF [68]. Patients with cardiogenic shock also have a higher frequency of CHIP mutations compared to stable HF patients, a finding that is also associated with worse survival and a pro-inflammatory cytokine profile [69]. The association between CHIP mutations and aberrant inflammatory cytokine profiles suggest that directed anti-inflammatory therapies may modify the observed effect of CHIP mutations on adverse cardiovascular and HF outcomes.

### The anti-inflammatory effects of guideline-directed HF therapy

**Fig. 2:**
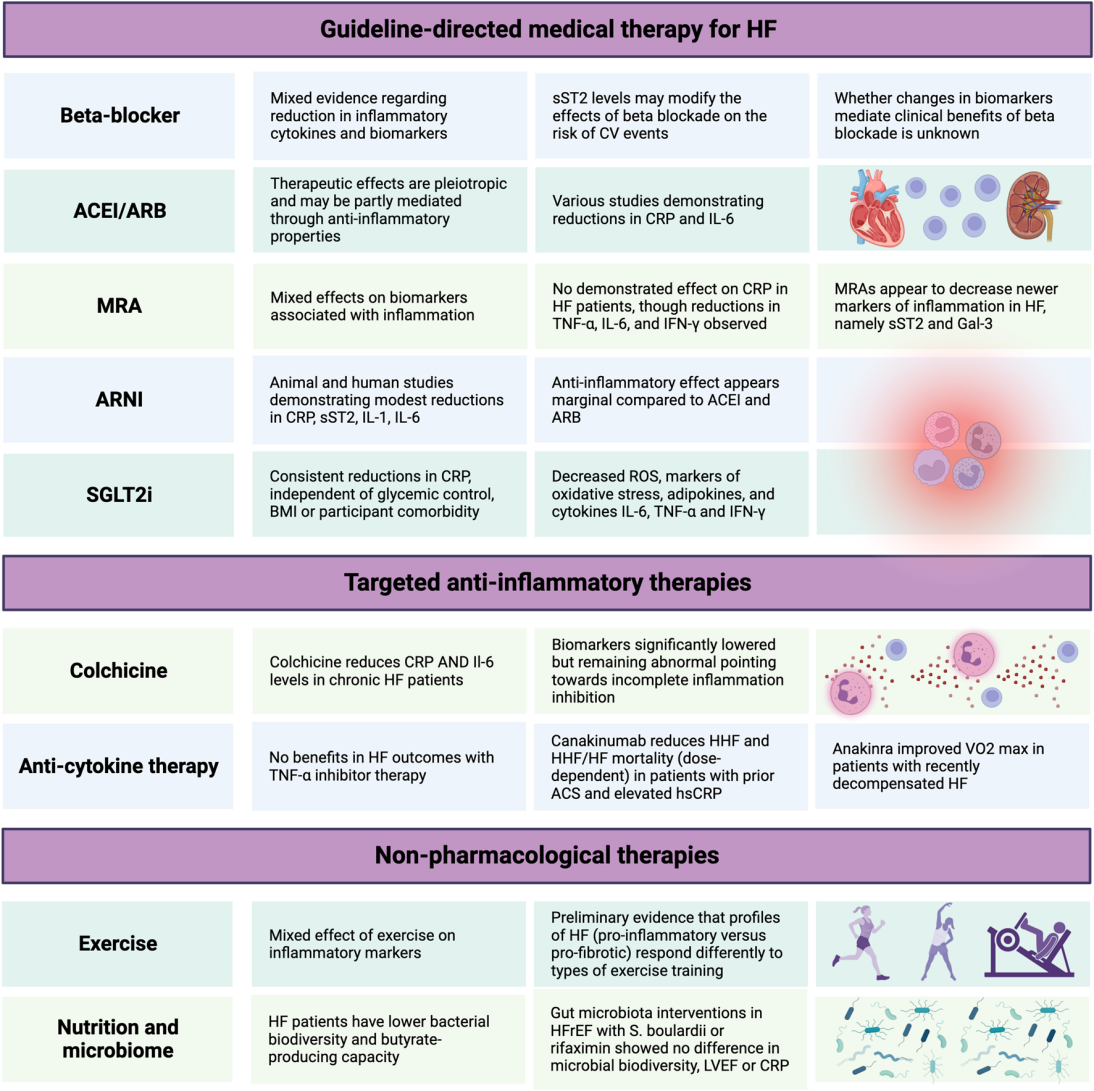
Therapies in Heart Failure and Inflammation. ACEI: angiotensin-converting enzyme inhibitor. *ACS* acute coronary syndrome, *ARB* angiotensin receptor blocker, *ARNI* angiotensin receptor neprilysin inhibitor, *CRP* C-reactive protein, *CV* cardiovascular, *GAL-3* galectin-3, *HF* heart failure, *HHF* heart failure hospitalization, *IFN* interferon, *IL* interleukin, *LVEF* left ventricular ejection fraction, *RAS* renin–angiotensin system, *SGLT2i* sodium glucose transporter 2 inhibitor, *sST2* soluble suppression of tumorigenesis-2, *TNF* tumor necrosis factor

The established clinical benefits of guideline-directed medical therapy (GDMT) in HF may in part be attributed to the immunomodulatory role of these agents. Their effects on inflammatory markers and circulating inflammatory cytokines, as well as the potential impacts of these specific effects on prognosis and clinical outcomes will be discussed (Fig. 2).

#### Beta-blocker therapy

While beta-blockers improve clinical status and LV function in the setting of HF and reduced ejection fraction, their anti-inflammatory effects are less apparent. Reductions in levels of circulating inflammatory markers with beta-blockade have been inconsistent and limited to non-randomized studies or those with small sample sizes [33, 70]. In one meta-analysis of randomized controlled trials with carvedilol, Tatli et al. reported reductions in plasma TNF-alpha, IL-2 and IL-6 accompanied by improvements in EF and HF severity in patients with dilated cardiomyopathy [71]. Similarly, Krum et al. reported reductions in TNF-alpha and IL-6 with carvedilol compared to placebo in participants with ischemic and non-ischemic CMP [72]. Subsequently, Toyoda and colleagues compared bisoprolol with carvedilol in HF patients and reported reductions in hs-CRP and markers of oxidative stress in both study groups [73]. The reports outlined above were from single-center studies with small to medium sample sizes, and whether these changes in biomarkers have been associated with a parallel improvement in LV function and /or a direct anti-inflammatory effect of beta-blocker therapy remains unknown.

The effects of beta-blockers on other biomarkers associated with surrogate markers of inflammation has also been described. Beta-blockers appear to increase or have neutral effects on NP in the short term, but consistently reduce NP in the longer term [74]. However, the degree to which these reductions are mediated by neurohumoral improvements versus anti-inflammatory effects has not been elucidated. Levels of sST2 and Gal-3 have been reported to modify the effects of beta-blockade on CV events and mortality events, though absolute number of events were small [75, 76]. With respect to the effect of changes in beta-blocker on sST2 levels, Gaggin and colleagues described an increase in sST2 levels after reduction in beta-blocker dosing [77]. In the same study, serial reductions in sST2 predicted a decrease in left ventricular end-diastolic index. The effect of beta-blockers on Gal-3 levels over the course of therapy is less clear, with human and animal studies demonstrating mixed effects [78, 79].

Despite their positive effect on clinical status, myocardial function, and reverse remodeling, beta-blockers have been associated with modest changes, if any, on pro-inflammatory markers.

#### Angiotensin-converting enzyme inhibitors and angiotensin receptor blockers

Through the blockade of angiotensin II which has pro-inflammatory and pro-fibrotic properties, angiotensin-converting inhibitors (ACEI) and angiotensin receptor blockers (ARB) reduce the production of ROS, pro-inflammatory cytokines, and adhesion molecules [80]. The therapeutic benefits of ACEI in patients with HF are likely pleiotropic and may in part be mediated through anti-inflammatory mechanisms. In a prospective observational study conducted in 507 patients with first-ever ischemic stroke, the use of ACEI was associated with a 2.6-fold decrease in median CRP levels and with a reduced 2-year CV risk [81]. These benefits were found to be independent of the lowering blood pressure effect of ACEI. In HFrEF patients, the addition of an ARB to ACEI and BB therapy was associated with a statistically significant decrease in hs-CRP and NT-proBNP at 6 months [82]. Also, the use of ACEI with high-dose enalapril in patients with congestive HF compared with healthy controls was associated with a statistically significant decrease in IL-6 bioactivity [83]. Overall, both ACEI and ARB show evidence of anti-inflammatory properties through their pleiotropic properties with demonstrated benefits in the HF population.

#### Angiotensin receptor neprilysin inhibitor

ARNI has also been described to harbor anti-inflammatory properties similar to ACEI and ARB. Goncalves et al. prospectively compared CRP values before and six months after sacubitril–valsartan therapy in HF patients with reduced ejection fraction [84]. After 6 months of treatment, more than two-thirds of patients had a reduction in CRP levels with a statistically significant reduction compared to baseline. Reductions in inflammatory cytokines with sacubitril–valsartan therapy have, however, been limited to animal models [85], PARADIGM-HF was the landmark trial demonstrating efficacy with sacubitril–valsartan in HF and reduced ejection fraction. While Gal-3 was elevated at baseline in PARADIGM-HF, there was no statistically significant difference between treatment groups with respect to changes in Gal-3 [86]. Baseline sST2 concentrations were found to have prognostic significance for the composite outcome of CV death or HF hospitalization for both treatment groups in PARADIGM-HF. Treatment with sacubitril–valsartan also led to a statistically significant decrease in sST2, a finding demonstrated in the PIONEER-HF trial as early as 1 week after randomization [87, 88]

These results suggest a significant effect of sacubitril–valsartan on subclinical inflammation, although its impact has been reported on a limited number of biomarkers. Nevertheless, the anti-inflammatory effect of sacubitril–valsartan appears marginal compared to other angiotensin modulating agents such as ACE inhibitors and ARB.

#### Mineralocorticoid receptor antagonists

Considering the role aldosterone plays in promoting cardiac inflammation and fibrosis, improvements in these deleterious pathways are believed to mediate the clinical benefits of MRAs in HF. This is supported by a secondary analysis of the EPHESUS trial suggesting that the survival benefits of eplerenone were independent of its diuretic and potassium-sparing effects [89]. Specifically, MRA is proposed to reduce oxidative stress, decrease ACE activity, and increase ACE2 activity along with Ang 1–7 [90].

Spironolactone has been shown to lower levels of NT-proBNP and BNP in HF patients, but no effect on CRP or uric acid was observed [91, 92]. In HF patients, spironolactone was found to decrease the production of TNF-α, IL-6, and INF-γ, which was found to be independent from its anti-mineralocorticoid and anti-androgen activities [93]. With respect to new markers in HF such as sST2 and Gal-3, their prognostic effects appear to be intact and independent of concomitant MRA therapy [94]. In animal models of acute MI and LV systolic dysfunction, treatment with an MRA was shown to result in lower levels of Gal-3 and sST2 compared to controls, a finding that was also correlated with lower expression of pro-inflammatory and fibrotic markers [95]. At least one study in participants with acute decompensated HF demonstrated reductions in sST2 with spironolactone within 3 days of initiation compared to placebo [96].

In summary, the evidence for MRAs’ anti-inflammatory properties in the literature is mixed. While there is clear evidence that MRAs lower biomarkers of the RAAS system and inflammatory cytokines, the literature failed to report a decrease in other traditional markers of inflammation that are used clinically such as CRP and uric acid. Additionally, MRAs seem to decrease newer markers of inflammation in HF, namely, sST2 and Gal-3.

#### Sodium glucose cotransporter 2 (SGLT2) inhibitors

Some of the improvements in HF and kidney outcomes mediated by SGLT2 inhibition have been attributed to anti-inflammatory pathways. Their systemic effects in addition to their hemodynamic benefits that have been previously described [97]. In a systematic review involving 23 heterogeneous studies with more than 1600 participants, 10 of 12 studies that measured CRP demonstrated significant reductions associated with SGLT2 inhibition [98]. Improvements in systemic and organ-level inflammation with SGLT2 inhibitors have been proposed to occur via reductions in oxidative stress, advanced glycolytic end products, ameliorations in cytokine/chemokine profiles, and improvement in adipose tissue function, as reflected by changes in associated biomarkers.

SGLT2 inhibition has been shown to decrease ROS generation in human coronary arterial endothelial cells and to reduce myocardial ROS and cardiac fibrosis in diabetic mice [99]. Additionally, adipose tissue contributes to inflammation through the release of adipokines including leptin, while reducing levels of the anti-inflammatory adipokine, adiponectin—these changes have been shown to be reversed after treatment with SGLT2 inhibition [100]. Beyond adipokines, SGLT2 inhibitors have also been shown to have favorable effects on pro-inflammatory cytokines including IL-6, TNF-α, and IFN-γ in several studies [98].

Mechanisms involved with reductions in inflammation at the level of cardiac tissue are less clear. Improvements in oxidative stress associated with ischemia reperfusion injury is one proposed mechanism. The nucleotide-binding oligomerization domain, leucine-rich repeat, and pyrin domain-containing 3 (NLRP3) inflammasome are also believed to play a mechanistic role in the pathogenesis and severity of HF in animal and human studies [101] Subsequent studies using animal HF models (and models of other organ failure) have demonstrated decreased activation of NLRP3 independent of diabetes status, offering another potential mechanism for improving inflammation in HF with SGLT2 inhibition [102]. Reductions in myocardial wall stress accompanying improvements in overall volume status in the setting of HF are also hypothesized to reduce cardiac inflammation. Experimental models have also demonstrated that SGLT2 inhibition activates nutrient deprivation signaling through the SIRT1/PGC-1α/ FGF21 pathway [103]. Activation of this pathway has been proposed to stimulate autophagy of cellular sources of oxidative and endoplasmic reticulum stress, alleviating a pro-inflammatory state and adverse cardiac remodeling [103].

#### Diuretics and decongestive therapies

Portal congestion in patients with HF has been associated with increased lymphocyte apoptosis from inflammatory cytokine release [104]. Congestion in the venous portal system also leads to bacterial endotoxin translocation from altered permeability of the gastrointestinal tract, which contributes to the immune system activation in HF patients [104, 105]. A low lymphocyte count ensues and lymphopenia in both acute and chronic HF has been associated with worse clinical outcomes [106, 107]. Additionally, lymphopenia has been associated with ultrasound surrogates of portal congestion and right ventricular failure in patients with signs and symptoms of HF with NYHA functional class II-IV managed with intravenous diuretic therapy [108]. While not strictly a GDMT, diuretics have been found to normalize concentrations of bacterial endotoxins and thus exert an indirect anti-inflammatory effect by decreasing gut edema [104]. The loop diuretic furosemide has been found to reduce levels of some inflammatory biomarkers in a dose-dependent manner, including TNF-α [109], IL-6, 8, and 10 [110]. Specifically in HF patients, furosemide has been shown to exert an anti-inflammatory effect by reducing TNF-α, IL-1β, and 6, in addition to natriuretic peptides [111]. The clinical significance of diuretic mediated reductions in inflammatory markers is unclear considering obvious confounders such as comorbidities, disease stage and severity, hyperactivation of the RAAS, and concomitant use of GDMT.

### Targeted anti-inflammatory therapy in HF

#### Colchicine and urate lowering therapy

Colchicine has several anti-inflammatory properties due to its inhibitory effects on microtubule polymerization and secondarily on the inflammasome. Interest in its application in CV disease has risen with the publication of multiple clinical trials over the last few years. Specifically, colchicine has been clearly demonstrated in two large, randomized trials (COLCOT and LoDoCo2) to be beneficial in reducing the risk of atherothrombotic CV events in patients with coronary artery disease [112, 113]. Post-acute myocardial infarction, reduced infarct size, and pro-inflammatory cytokine levels were demonstrated in mice after a short-term treatment of colchicine post-permanent ligation of the left anterior coronary artery [114]. In this study, survival rate and left ventricular end-diastolic diameter were improved, with an associated reduction in natriuretic peptide expression. In contrast, the effect of colchicine in chronic HF was only studied in a small placebo-controlled study of 267 patients followed for 6 months [115]. Although CRP and IL-6 levels were significantly reduced after treatment with colchicine in HF, there were no differences observed with respect to the primary outcome of NYHA functional status. While inflammatory markers were significantly lowered with colchicine, they remained abnormal pointing toward incomplete inhibition of the inflammatory process. The role of colchicine in HF has therefore yet to be established [116]. The clinical impact of colchicine in patients with various phenotypes and severity of HF remains subject to further investigations. The COLpEF trial (NCT04857931) is one such planned study of colchicine in the patients with HFpEF, with a proposed outcomes including CRP, NT-proBNP, as well as changes in functional status and echocardiographic parameters.

As discussed above, elevated uric acid levels have been associated with worse prognosis in patients with HF, although studies assessing the effect of uric acid reduction with xanthine oxidase inhibitors such as the OPT-CHF [117] and EXACT-HF [118] trials failed to demonstrate any significant clinical benefit of the chronic administration of allopurinol in these patients.

#### Therapies inhibiting cytokine pathways

Agents targeting inflammatory cytokine pathways have largely been studied in the context of preventing atherosclerotic CV outcomes. Pertinent examples include the IL-1β inhibitor canakinumab in the CANTOS trial [119], low-dose methotrexate in the CIRT trial [120], and comparing the vascular safety of the IL-6 inhibitor tocilizumab with the TNF-α inhibitor etanercept in the setting of rheumatoid arthritis in the ENTRACTE trial [121]. The TNF-α inhibitor infliximab was studied in a pilot placebo-controlled study (ATTACH Trial) of patients with moderate to severe HF for 28 weeks [122]. Despite reducing CRP and IL-6 levels, infliximab failed to improve clinical status and appeared to be associated with adverse events with the highest dose. Shortly after, the RENEWAL study examined the effect of the TNF-α inhibitor etanercept in HFrEF patients and failed to demonstrate any benefit on mortality or hospitalization for HF (HHF) [123]. The effect of the IL-1 beta inhibitor canakinumab on HF outcomes was studied in a prespecified secondary analysis of the CANTOS trial in participants with prior ACS and elevated hs-CRP [124]. In this exploratory analysis, canakinumab was noted to reduce HHF and the composite of HHF and HF mortality in a dose-dependent manner. The same pathway was targeted using the IL-1 receptor antagonist anakinra in a series of smaller randomized trials in HF patients [125]. In recently decompensated HF, anakinra improved peak oxygen consumption (VO2 max) at 12 weeks compared to placebo [126]. Furthermore, in patients with ST-elevation myocardial infarction (STEMI), 14-day treatment with anakinra reduced hs-CRP levels as well as the incidence of new-onset HF or HF hospitalization at 1-year after the STEMI [127]. Longer and larger studies are required to examine the impact of anakinra on harder HF outcomes. Taken together, these exploratory studies do appear to suggest a beneficial role of some anti-inflammatory therapies in HF, particularly those targeting interleukins proximal to the IL-6 cascade [128]. Indeed, ziltivekimab, a human monoclonal antibody against IL-6, has been shown in the RESCUE study to reduce high-sensitivity CRP in participants at high atherosclerotic risk [129]. Its effects on CV, heart failure, and mortality outcomes in participants with HF and at high CV risk are being studied in the HERMES (NCT05636176) and ZEUS (NCT05021835) trials, respectively.

### Non-pharmacological interventions

#### Exercise

Several small studies have demonstrated the benefits of exercise on systemic inflammation and larger population studies have shown an inverse relationship between physical activity and biomarker levels of systemic inflammation [130]. The current literature is limited in randomized controlled trials assessing the effect of exercise training on inflammation, especially in the HF population, and results drawn from those studies are often inconclusive. Exercise has also been shown to reduce circulating levels of biomarkers specifically in HF patients, including TNF-α, IL-6, and CRP [131]. On the contrary, a recent systematic review failed to demonstrate any benefit on inflammatory biomarkers with exercise training in patients with congestive HF [132]. Interestingly, a single-center multi-arm controlled clinical trial comparing the effects of a 12-week aerobic exercise training program on functional capacity in patients with HF demonstrated that different inflammatory profiles (pro-inflammatory versus pro-fibrotic) respond differently to types of exercise training [133]. Future randomized controlled studies need to improve our understanding regarding the role and potential benefits of exercise training with respect to inflammation in the HF population.

#### Nutrition and microbiome interventions

Multiple studies have described altered gut microbiomes in patients with HF, a finding that may have prognostic relevance and be amenable to therapeutic interventions [134]. One possible way the gut–heart axis affects HF outcomes is through decreased mucosal integrity from intestinal ischemia or edema—subsequent leakage of bacterial components has been hypothesized to contribute to systemic inflammation and worse outcomes [135]. Diet is one potential intervention that has been proposed to favorably alter gut microbiota in HF patients. In a cross-sectional analysis, Mayerhofer and colleagues reported that HF patients had lower bacterial biodiversity and a lower butyrate-producing capacity, integral to maintaining the gut–blood barrier. This finding was specifically associated with lower fiber intake, a modifiable risk factor [136]. The same group also studied modification of gut microbiota in HFrEF patients with *Saccharomyces boulardii* or rifaximin for three months on top of standard HF therapy [137]. However, neither therapy demonstrated differences in gut microbial biodiversity, LVEF, or CRP. Given the compelling preclinical data describing the role of the gut–heart axis in HF, targeted studies in select HF population may be needed to explore therapeutic interventions.

HF and other conditions associated with inflammatory activation also tend to be associated with iron deficiency. Treatment of iron deficiency in HF populations has been demonstrated in clinical trials and meta-analyses to improve quality of life, rates of HF hospitalizations, and mortality [138]. FAIR-HF2 (NCT03036462) is an ongoing prospective trial designed to explore the effects on intravenous iron on HF hospitalizations and CV death.

## Conclusion

Inflammation plays a significant role in the pathogenesis of the HF syndrome, with maladaptive responses within the innate and adaptive immune systems contributing to adverse cardiac remodeling, fibrosis, and perturbed vascular and extra-cardiac organ physiology. There are most likely multiple mechanisms involved that remain poorly understood. In this review, we highlighted key biomarkers in HF and how improvements in inflammatory pathways may mediate clinical benefits of existing GDMT for HF, as well as novel therapies in the pipeline targeting HF and inflammation. Biomarkers may also have a role to play in the identification of patients that may benefit further from augmentation or optimization of HF therapy, an approach that may eventually guide future trial design and be incorporated into the classification of HF.

## Data Availability

All data supporting the findings from this study are available from the corresponding author upon reasonable request.

## References

[1] Liberale L, Montecucco F, Tardif JC, Libby P, Camici GG (2020). Inflamm-ageing: the role of inflammation in age-dependent cardiovascular disease. Eur Heart J.

[2] Pfisterer M (2009). BNP-guided vs symptom-guided heart failure therapy: the Trial of Intensified vs Standard Medical Therapy in Elderly Patients With Congestive Heart Failure (TIME-CHF) randomized trial. JAMA.

[3] O'Connor CM (2011). Effect of nesiritide in patients with acute decompensated heart failure. N Engl J Med.

[4] Anand IS (2005). C-reactive protein in heart failure: prognostic value and the effect of valsartan. Circulation.

[5] White M (2013). Cardiac signaling molecules and plasma biomarkers after cardiac transplantation: impact of tacrolimus versus cyclosporine. J Heart Lung Transplant.

[6] Tromp J (2017). Biomarker profiles of acute heart failure patients with a mid-range ejection fraction. JACC Heart Fail.

[7] Boulet J, Massie E, Rouleau J-L (2021). Heart failure with midrange ejection fraction—what is it, if anything?. Can J Cardiol.

[8] Paulus WJ, Tschope C (2013). A novel paradigm for heart failure with preserved ejection fraction: comorbidities drive myocardial dysfunction and remodeling through coronary microvascular endothelial inflammation. J Am Coll Cardiol.

[9] Paulus WJ, Zile MR (2021). From systemic inflammation to myocardial fibrosis: the heart failure with preserved ejection fraction paradigm revisited. Circ Res.

[10] Dick SA, Epelman S (2016). Chronic heart failure and inflammation: what do we really know?. Circ Res.

[11] Murphy SP, Kakkar R, McCarthy CP, Januzzi JL (2020). Inflammation in heart failure: JACC state-of-the-art review. J Am Coll Cardiol.

[12] Vasan RS (2003). Inflammatory markers and risk of heart failure in elderly subjects without prior myocardial infarction: the Framingham Heart Study. Circulation.

[13] Pellicori P (2020). High-sensitivity C-reactive protein in chronic heart failure: patient characteristics, phenotypes, and mode of death. Cardiovasc Res.

[14] Givertz MM (2018). Treating gout in patients with cardiovascular disease: mutual benefit or unintended consequences?. J Am Coll Cardiol.

[15] Anker SD (2003). Uric acid and survival in chronic heart failure: validation and application in metabolic, functional, and hemodynamic staging. Circulation.

[16] Ruocco G, Palazzuoli A (2019). Hyperuricemia in US population with heart failure: causal or incidental bystander?. Cardiorenal Med.

[17] Salvetti M, Paini A, Agabiti-Rosei C, Muiesan ML (2016). Uric acid and cardiovascular disease an update. Eur Cardiol Rev.

[18] Roig E, Perez-Villa F, Morales M, Jimenez W, Orus J, Heras M, Sanz G (2000). Clinical implications of increased plasma angiotensin II despite ACE inhibitor therapy in patients with congestive heart failure. Eur Heart J.

[19] Epelman S, Shrestha K, Troughton RW, Francis GS, Sen S, Klein AL, Tang WH (2009). Soluble angiotensin-converting enzyme 2 in human heart failure: relation with myocardial function and clinical outcomes. J Card Fail.

[20] Wang K, Basu R, Poglitsch M, Bakal JA, Oudit GY (2020). Elevated angiotensin 1–7/angiotensin ii ratio predicts favorable outcomes in patients with heart failure. Circ Heart Fail.

[21] Frangogiannis NG (2014). The inflammatory response in myocardial injury, repair, and remodelling. Nat Rev Cardiol.

[22] Vitiello D, Neagoe P, Sirois MG, White M (2013). Characterization and modulation of neutrophils inflammatory response in advanced heart failure. Can J Cardiol.

[23] Vulesevic B, Sirois MG, Allen BG, de Denus S, White M (2018). Subclinical inflammation in heart failure: a neutrophil perspective. Can J Cardiol.

[24] Arbel Y (2012). Neutrophil/lymphocyte ratio is related to the severity of coronary artery disease and clinical outcome in patients undergoing angiography. Atherosclerosis.

[25] Curran FM (2021). Neutrophil-to-lymphocyte ratio and outcomes in patients with new-onset or worsening heart failure with reduced and preserved ejection fraction. ESC Heart Fail.

[26] Basso C (2022). Myocarditis. N Engl J Med.

[27] Chaar D, Dumont B, Vulesevic B, Neagoe PE, Rakel A, Sirois MG, White M (2021). Neutrophils pro-inflammatory and anti-inflammatory cytokine release in patients with heart failure and reduced ejection fraction. ESC Heart Fail.

[28] Fish-Trotter H (2020). Inflammation and circulating natriuretic peptide levels. Circ Heart Fail.

[29] McKechnie DG, Papacosta AO, Lennon LT, Welsh P, Whincup PH, Wannamethee SG (2021). Inflammatory markers and incident heart failure in older men: the role of NT-proBNP. Biomark Med.

[30] Deswal A, Petersen NJ, Feldman AM, Young JB, White BG, Mann DL (2001). Cytokines and cytokine receptors in advanced heart failure: an analysis of the cytokine database from the Vesnarinone trial (VEST). Circulation.

[31] Torre-Amione G, Kapadia S, Benedict C, Oral H, Young JB, Mann DL (1996). Proinflammatory cytokine levels in patients with depressed left ventricular ejection fraction: a report from the studies of left ventricular dysfunction (SOLVD). J Am Coll Cardiol.

[32] Rauchhaus M (2000). Plasma cytokine parameters and mortality in patients with chronic heart failure. Circulation.

[33] Gullestad L, Ueland T, Brunsvig A, Kjekshus J, Simonsen S, Froland SS, Aukrust P (2001). Effect of metoprolol on cytokine levels in chronic heart failure—a substudy in the Metoprolol Controlled-Release Randomised Intervention Trial in Heart Failure (MERIT-HF). Am Heart J.

[34] Segiet OA, Piecuch A, Mielanczyk L, Michalski M, Nowalany-Kozielska E (2019). Role of interleukins in heart failure with reduced ejection fraction. Anatol J Cardiol.

[35] Van Tassell BW, Seropian IM, Toldo S, Mezzaroma E, Abbate A (2013). Interleukin-1beta induces a reversible cardiomyopathy in the mouse. Inflamm Res.

[36] Testa M, Yeh M, Lee P, Berman JW, Lejemtel TH, Fanelli R, Loperfido F (1996). Circulating levels of cytokines and their endogenous modulators in patients with mild to severe congestive heart failure due to coronary artery disease or hypertension. J Am Coll Cardiol.

[37] Ridker PM, MacFadyen JG, Thuren T, Libby P (2020). Residual inflammatory risk associated with interleukin-18 and interleukin-6 after successful interleukin-1β inhibition with canakinumab: further rationale for the development of targeted anti-cytokine therapies for the treatment of atherothrombosis. Eur Heart J.

[38] Markousis-Mavrogenis G (2019). The clinical significance of interleukin-6 in heart failure: results from the BIOSTAT-CHF study. Eur J Heart Fail.

[39] Chia YC (2021). Interleukin 6 and development of heart failure with preserved ejection fraction in the general population. J Am Heart Assoc.

[40] Nymo SH (2014). Inflammatory cytokines in chronic heart failure: interleukin-8 is associated with adverse outcome. Results from CORONA. Eur J Heart Fail.

[41] Caselli C (2013). IL-33/ST2 pathway and classical cytokines in end-stage heart failure patients submitted to left ventricular assist device support: a paradoxic role for inflammatory mediators?. Mediators Inflamm.

[42] Ezekowitz JA (2017). Comprehensive update of the Canadian cardiovascular society guidelines for the management of heart failure. Can J Cardiol.

[43] Braunwald E (2008). Biomarkers in heart failure. N Engl J Med.

[44] Aimo A, Januzzi JL, Bayes-Genis A, Vergaro G, Sciarrone P, Passino C, Emdin M (2019). Clinical and prognostic significance of sST2 in heart failure: JACC review topic of the week. J Am Coll Cardiol.

[45] Pascual-Figal DA, Januzzi JL (2015). The biology of ST2: the International ST2 consensus panel. Am J Cardiol.

[46] Seki K, Sanada S, Kudinova AY, Steinhauser ML, Handa V, Gannon J, Lee RT (2009). Interleukin-33 prevents apoptosis and improves survival after experimental myocardial infarction through ST2 signaling. Circ Heart Fail.

[47] Sanada S, Hakuno D, Higgins LJ, Schreiter ER, McKenzie AN, Lee RT (2007). IL-33 and ST2 comprise a critical biomechanically induced and cardioprotective signaling system. J Clin Investig.

[48] Emdin M (2018). sST2 Predicts outcome in chronic heart failure beyond NT-proBNP and high-sensitivity troponin T. J Am Coll Cardiol.

[49] Sanders-van Wijk S (2015). Circulating biomarkers of distinct pathophysiological pathways in heart failure with preserved vs. reduced left ventricular ejection fraction. Eur J Heart Fail.

[50] Parikh RH, Seliger SL, Christenson R, Gottdiener JS, Psaty BM, deFilippi CR (2016). Soluble ST2 for prediction of heart failure and cardiovascular death in an elderly, community-dwelling population. J Am Heart Assoc.

[51] Bayes-Genis A (2014). Head-to-head comparison of 2 myocardial fibrosis biomarkers for long-term heart failure risk stratification: ST2 versus galectin-3. J Am Coll Cardiol.

[52] Mueller T, Gegenhuber A, Leitner I, Poelz W, Haltmayer M, Dieplinger B (2016). Diagnostic and prognostic accuracy of galectin-3 and soluble ST2 for acute heart failure. Clin Chim Acta.

[53] Johannes L, Jacob R, Leffler H (2018). Galectins at a glance. J Cell Sci.

[54] Sharma UC (2004). Galectin-3 marks activated macrophages in failure-prone hypertrophied hearts and contributes to cardiac dysfunction. Circulation.

[55] Besler C (2017). Plasma and cardiac galectin-3 in patients with heart failure reflects both inflammation and fibrosis: implications for its use as a biomarker. Circ Heart Fail.

[56] Lok DJ, Van Der Meer P, de la Porte PW, Lipsic E, Van Wijngaarden J, Hillege HL, van Veldhuisen DJ (2010). Prognostic value of galectin-3, a novel marker of fibrosis, in patients with chronic heart failure: data from the DEAL-HF study. Clin Res Cardiol.

[57] Lok DJ (2013). Galectin-3 is an independent marker for ventricular remodeling and mortality in patients with chronic heart failure. Clin Res Cardiol.

[58] van Kimmenade RR (2006). Utility of amino-terminal pro-brain natriuretic peptide, galectin-3, and apelin for the evaluation of patients with acute heart failure. J Am College Cardiol.

[59] Poniatowski ŁA, Wojdasiewicz P, Gasik R, Szukiewicz D (2015). Transforming growth factor Beta family: insight into the role of growth factors in regulation of fracture healing biology and potential clinical applications. Mediators Inflamm.

[60] Sharma A (2017). Utility of growth differentiation factor-15, a marker of oxidative stress and inflammation, in chronic heart failure: insights from the HF-ACTION study. JACC Heart Failure.

[61] Daniels LB, Clopton P, Laughlin GA, Maisel AS, Barrett-Connor E (2011). Growth-differentiation factor-15 is a robust, independent predictor of 11-year mortality risk in community-dwelling older adults: the Rancho Bernardo Study. Circulation.

[62] Dallmeier D, Brenner H, Mons U, Rottbauer W, Koenig W, Rothenbacher D (2016). Growth differentiation factor 15, its 12-month relative change, and risk of cardiovascular events and total mortality in patients with stable coronary heart disease: 10-year follow-up of the KAROLA atudy. Clin Chem.

[63] Bouabdallaoui N (2018). Growth differentiation factor-15 is not modified by sacubitril/valsartan and is an independent marker of risk in patients with heart failure and reduced ejection fraction: the PARADIGM-HF trial. Eur J Heart Fail.

[64] Sawalha K, Norgard NB, Drees BM, López-Candales A (2003). Growth differentiation factor 15 (GDF-15), a new biomarker in heart failure management. Curr Heart Failure Rep.

[65] Libby P, Ebert BL (2018). CHIP (Clonal Hematopoiesis of Indeterminate Potential): potent and newly recognized contributor to cardiovascular risk. Circulation.

[66] Mockel M (2020). Improve Management of acute heart failure with ProcAlCiTonin in EUrope: results of the randomized clinical trial IMPACT EU biomarkers in cardiology (BIC) 18. Eur J Heart Fail.

[67] Yu B (2021). Supplemental association of clonal hematopoiesis with incident heart failure. J Am Coll Cardiol.

[68] Dorsheimer L (2019). Association of mutations contributing to clonal hematopoiesis with prognosis in chronic ischemic heart failure. JAMA Cardiol.

[69] Scolari FL (2022). Clonal haematopoiesis is associated with higher mortality in patients with cardiogenic shock. Eur J Heart Fail.

[70] Ohtsuka T (2001). Effect of beta-blockers on circulating levels of inflammatory and anti-inflammatory cytokines in patients with dilated cardiomyopathy. J Am Coll Cardiol.

[71] Tatli E, Kurum T (2005). A controlled study of the effects of carvedilol on clinical events, left ventricular function and proinflammatory cytokines levels in patients with dilated cardiomyopathy. Can J Cardiol.

[72] Kurum T, Tatli E, Yuksel M (2007). Effects of carvedilol on plasma levels of pro-inflammatory cytokines in patients with ischemic and nonischemic dilated cardiomyopathy. Tex Heart Inst J.

[73] Toyoda S (2020). Effects of carvedilol vs bisoprolol on inflammation and oxidative stress in patients with chronic heart failure. J Cardiol.

[74] Troughton RW, Richards AM, Yandle TG, Frampton CM, Nicholls MG (2007). The effects of medications on circulating levels of cardiac natriuretic peptides. Ann Med.

[75] Gaggin HK, Motiwala S, Bhardwaj A, Parks KA, Januzzi JL (2013). Soluble concentrations of the interleukin receptor family member ST2 and beta-blocker therapy in chronic heart failure. Circ Heart Fail.

[76] Sanders-van Wijk S (2016). Interaction of galectin-3 concentrations with the treatment effects of beta-blockers and RAS Blockade in patients with systolic heart failure: a derivation-validation study from TIME-CHF and GISSI-HF. Clin Chem.

[77] Gaggin HK (2014). Head-to-head comparison of serial soluble ST2, growth differentiation factor-15, and highly-sensitive troponin T measurements in patients with chronic heart failure. JACC Heart Fail.

[78] Motiwala SR (2013). Serial measurement of galectin-3 in patients with chronic heart failure: results from the ProBNP Outpatient Tailored Chronic Heart Failure Therapy (PROTECT) study. Eur J Heart Fail.

[79] Nguyen MN (2018). Mechanisms responsible for increased circulating levels of galectin-3 in cardiomyopathy and heart failure. Sci Rep.

[80] Brasier AR, Recinos A, Eledrisi MS (2002). Vascular inflammation and the renin-angiotensin system. Arterioscler Thromb Vasc Biol.

[81] Di Napoli M, Papa F (2003). Angiotensin-converting enzyme inhibitor use is associated with reduced plasma concentration of C-reactive protein in patients with first-ever ischemic stroke. Stroke.

[82] White M (2007). Effects of combined candesartan and ACE inhibitors on BNP, markers of inflammation and oxidative stress, and glucose regulation in patients with symptomatic heart failure. J Cardiac Fail.

[83] Gullestad L (1999). Effect of high- versus low-dose angiotensin converting enzyme inhibition on cytokine levels in chronic heart failure. J Am Coll Cardiol.

[84] Valentim Goncalves A (2020). C-reactive protein reduction with sacubitril-valsartan treatment in heart failure patients. Am J Cardiovasc Dis.

[85] Mohany M, Alanazi AZ, Alqahtani F, Belali OM, Ahmed MM, Al-Rejaie SS (2020). LCZ696 mitigates diabetic-induced nephropathy through inhibiting oxidative stress, NF-κB mediated inflammation and glomerulosclerosis in rats. PeerJ.

[86] McMurray JJV (2014). Angiotensin–neprilysin inhibition versus enalapril in heart failure. N Engl J Med.

[87] Morrow DA (2019). Cardiovascular biomarkers in patients with acute decompensated heart failure randomized to sacubitril-valsartan or enalapril in the PIONEER-HF trial. Eur Heart J.

[88] Verma S (2019). Effect of empagliflozin on left ventricular mass in patients with type 2 diabetes mellitus and coronary artery disease. Circulation.

[89] Rossignol P, Menard J, Fay R, Gustafsson F, Pitt B, Zannad F (2011). Eplerenone survival benefits in heart failure patients post-myocardial infarction are independent from its diuretic and potassium-sparing effects. Insights from an EPHESUS (Eplerenone Post-Acute Myocardial Infarction Heart Failure Efficacy and Survival Study) substudy. J Am Coll Cardiol.

[90] Keidar S (2005). Mineralocorticoid receptor blocker increases angiotensin-converting enzyme 2 activity in congestive heart failure patients. Circ Res.

[91] Myhre PL (2020). Mechanistic effects of spironolactone on cardiovascular and renal biomarkers in heart failure with preserved ejection fraction: a TOPCAT biorepository study. Circ Heart Fail.

[92] Godfrey V, Farquharson CA, Macdonald JE, Yee KM, Struthers AD (2007). Effect of spironolactone on C-reactive protein levels in patients with heart disease. Int J Cardiol.

[93] Hansen PR, Rieneck K, Bendtzen K (2004). Spironolactone inhibits production of proinflammatory cytokines by human mononuclear cells. Immunol Lett.

[94] Barutaut M (2020). sST2 adds to the prognostic value of Gal-3 and BNP in chronic heart failure. Acta Cardiol.

[95] Lax A (2015). Mineralocorticoid receptor antagonists modulate galectin-3 and interleukin-33/ST2 signaling in left ventricular systolic dysfunction after acute myocardial infarction. JACC Heart Fail.

[96] Wasyanto T, Mufidah A (2019). Effect of early spironolactone on the ST2 level and clinical changes in acute decompensated heart failure patients. Indonesian J Med.

[97] Liu H, Sridhar VS, Boulet J, Dharia A, Khan A, Lawler PR, Cherney DZI (2021). Cardiorenal protection with SGLT2 inhibitors in patients with diabetes mellitus: from biomarkers to clinical outcomes in heart failure and diabetic kidney disease. Metabolism.

[98] Bray JJH, Foster-Davies H, Stephens JW (2020). A systematic review examining the effects of sodium-glucose cotransporter-2 inhibitors (SGLT2is) on biomarkers of inflammation and oxidative stress. Diabetes Res Clin Pract.

[99] Li C (2019). SGLT2 inhibition with empagliflozin attenuates myocardial oxidative stress and fibrosis in diabetic mice heart. Cardiovasc Diabetol.

[100] Garvey WT (2018). Effects of canagliflozin versus glimepiride on adipokines and inflammatory biomarkers in type 2 diabetes. Metabolism.

[101] Butts B, Gary RA, Dunbar SB, Butler J (2015). The importance of NLRP3 inflammasome in heart failure. J Card Fail.

[102] Byrne NJ (2020). Empagliflozin blunts worsening cardiac dysfunction associated with reduced NLRP3 (Nucleotide-Binding Domain-Like Receptor Protein 3) inflammasome activation in heart failure. Circ Heart Fail.

[103] Packer M (2020). Cardioprotective effects of sirtuin-1 and its downstream effectors: potential role in mediating the heart failure Benefits of SGLT2 (Sodium-Glucose Cotransporter 2) inhibitors. Circ Heart Fail.

[104] Niebauer J (1999). Endotoxin and immune activation in chronic heart failure: a prospective cohort study. Lancet.

[105] Sandek A (2007). Altered intestinal function in patients with chronic heart failure. J Am Coll Cardiol.

[106] Milo-Cotter O (2010). Low lymphocyte ratio as a novel prognostic factor in acute heart failure: results from the pre-RELAX-AHF Study. Cardiology.

[107] Vaduganathan M (2012). Predictive value of low relative lymphocyte count in patients hospitalized for heart failure with reduced ejection fraction: insights from the EVEREST trial. Circ Heart Fail.

[108] Bouabdallaoui N, Sirois MG, Beaubien-Souligny W, Denault AY, Rouleau JL (2020). Lymphocytopenia during hospitalization for acute heart failure and its relationship with portal congestion and right ventricular function. J Card Fail.

[109] Sheikhi A, Jaberi Y, Esmaeilzadeh A, Khani M, Moosaeefard M, Shafaqatian M (2007). The effect of cardiovascular drugs on pro-inflammatory cytokine secretion and natural killer activity of peripheral blood mononuclear cells of patients with chronic heart failure in vitro. Pak J Biol Sci.

[110] Prandota J (2002). Furosemide: progress in understanding its diuretic, anti-inflammatory, and bronchodilating mechanism of action, and use in the treatment of respiratory tract diseases. Am J Ther.

[111] Tuttolomondo A (2011). Changes in natriuretic peptide and cytokine plasma levels in patients with heart failure, after treatment with high dose of furosemide plus hypertonic saline solution (HSS) and after a saline loading. Nutr Metab Cardiovasc Dis.

[112] Tardif JC (2019). Efficacy and safety of low-dose colchicine after myocardial infarction. N Engl J Med.

[113] Nidorf SM (2020). Colchicine in patients with chronic coronary disease. N Engl J Med.

[114] Fujisue K (2017). Colchicine improves survival, left ventricular remodeling, and chronic cardiac function after acute myocardial infarction. Circ J.

[115] Deftereos S (2014). Anti-inflammatory treatment with colchicine in stable chronic heart failure: a prospective, randomized study. JACC Heart Fail.

[116] Hemkens LG (2016). Colchicine for prevention of cardiovascular events. The Cochrane Database System Rev.

[117] Hare JM (2008). Impact of oxypurinol in patients with symptomatic heart failure. Results of the OPT-CHF study. J Am Coll Cardiol.

[118] Givertz MM (2015). Effects of xanthine oxidase inhibition in hyperuricemic heart failure patients: the xanthine oxidase inhibition for hyperuricemic heart failure patients (EXACT-HF) Study. Circulation.

[119] Ridker PM (2017). Antiinflammatory therapy with canakinumab for atherosclerotic disease. N Engl J Med.

[120] Ridker PM (2019). Low-dose methotrexate for the prevention of atherosclerotic events. N Engl J Med.

[121] Giles JT (2020). Cardiovascular safety of tocilizumab versus etanercept in rheumatoid arthritis: a randomized controlled trial. Arthritis Rheumatol.

[122] Chung ES, Packer M, Lo KH, Fasanmade AA, Willerson JT, Anti TNFTACHFI (2003). Randomized, double-blind, placebo-controlled, pilot trial of infliximab, a chimeric monoclonal antibody to tumor necrosis factor-alpha, in patients with moderate-to-severe heart failure: results of the anti-TNF Therapy Against Congestive Heart Failure (ATTACH) trial. Circulation.

[123] Mann DL (2004). Targeted anticytokine therapy in patients with chronic heart failure: results of the Randomized Etanercept Worldwide Evaluation (RENEWAL). Circulation.

[124] Everett BM (2019). Anti-inflammatory therapy with canakinumab for the prevention of hospitalization for heart failure. Circulation.

[125] Van Tassell BW (2018). IL-1 Blockade in patients with heart failure with preserved ejection fraction. Circ Heart Fail.

[126] Van Tassell BW (2017). Interleukin-1 blockade in recently decompensated systolic heart failure: results from REDHART (Recently Decompensated Heart Failure Anakinra Response Trial). Circ Heart Fail.

[127] Abbate A (2020). Interleukin-1 blockade inhibits the acute inflammatory response in patients with ST-segment-elevation myocardial infarction. J Am Heart Assoc.

[128] Ridker PM, Rane M (2021). Interleukin-6 signaling and anti-interleukin-6 therapeutics in cardiovascular disease. Circ Res.

[129] Ridker PM (2021). IL-6 inhibition with ziltivekimab in patients at high atherosclerotic risk (RESCUE): a double-blind, randomised, placebo-controlled, phase 2 trial. The Lancet.

[130] Beavers KM, Brinkley TE, Nicklas BJ (2010). Effect of exercise training on chronic inflammation. Clin Chim Acta.

[131] Smart NA, Steele M (2011). The effect of physical training on systemic proinflammatory cytokine expression in heart failure patients: a systematic review. Congest Heart Fail.

[132] Pearson MJ, Mungovan SF, Smart NA (2018). Effect of aerobic and resistance training on inflammatory markers in heart failure patients: systematic review and meta-analysis. Heart Fail Rev.

[133] Fernandes-Silva MM (2017). Inflammatory biomarkers and effect of exercise on functional capacity in patients with heart failure: Insights from a randomized clinical trial. Eur J Prev Cardiol.

[134] Schiattarella GG (2017). Gut microbe-generated metabolite trimethylamine-N-oxide as cardiovascular risk biomarker: a systematic review and dose-response meta-analysis. Eur Heart J.

[135] Frantz S (2018). The innate immune system in chronic cardiomyopathy: a European Society of Cardiology (ESC) scientific statement from the Working Group on Myocardial Function of the ESC. Eur J Heart Fail.

[136] Mayerhofer CCK (2020). Low fibre intake is associated with gut microbiota alterations in chronic heart failure. ESC Heart Fail.

[137] Awoyemi A (2021). Rifaximin or *Saccharomyces boulardii* in heart failure with reduced ejection fraction: results from the randomized GutHeart trial. EBioMedicine.

[138] von Haehling S, Ebner N, Evertz R, Ponikowski P, Anker SD (2019). Iron deficiency in heart failure: an overview. JACC Heart Fail.

